# RPA-CRISPR/Cas12a-based detection of *Pasteurella multocida*: establishment and initial application

**DOI:** 10.3389/fvets.2025.1730229

**Published:** 2026-01-14

**Authors:** Chaoqun Yan, Xiaozhen Li, Rulong Chen, Quanxin Wu, Youquan Zhuang, Na Li, Xuelian Ma, Yuefeng Chu, Huijun Shi, Qiang Fu, Li Yang

**Affiliations:** 1 College of Animal Medicine, Xinjiang Agricultural University, Urumqi, China; 2 Xinjiang Key Laboratory of New Drug Research and Development for Herbivorous Animals, Urumqi, China; 3 Urumqi Customs Technology Center, Urumqi, China; 4 Xinjiang Tianlai Agriculture and Animal Husbandry Group Co., Ltd., Bole, China; 5 Chinese Academy of Agricultural Sciences, Lanzhou Veterinary Research Institute, Lanzhou, China; 6 Zhejiang University School of Medicine, Hangzhou, China

**Keywords:** *Pasteurella multocida*, RPA, CRISPR/Cas12a, visual detection, PCR

## Abstract

**Introduction:**

*Pasteurella multocida* (Pm) is a major pathogen that causes respiratory diseases in sheep, leading to high morbidity, high mortality, and significant economic losses. Current diagnostic methods, such as bacterial isolation, ELISA, and PCR, are limited by low throughput, complex procedures, and reliance on specialized equipment, making them unsuitable for field use.

**Methods:**

In this study, we developed a rapid, visual, and sensitive method for detecting Pm by combining recombinase polymerase amplification (RPA) with CRISPR/Cas12a. The PCR method based on kmt1 is the “gold standard” for studying Pm. So this assay targeted the kmt1 gene and was optimized for primer selection, reaction conditions, and crRNA/Cas12a ratios. Specificity verification was conducted through common respiratory pathogens, and sensitivity verification was carried out using plasmid dilution solutions.

**Results:**

The method showed a detection limit of 5 × 10^−1^ copies/μL, and the reactions were completed within 30 min. When applied to 102 clinical samples, the RPA-CRISPR/Cas12a assay yielded a positive rate of 40.20% (41/102), which was 4.1 times higher than that of PCR. This assay offers a promising tool for rapid and instrument-free detection of Pm in frontline clinical settings.

## 1 Introduction

*Pasteurella multocida* (Pm) is a gram-negative pathogenic bacterium that can cause a wide range of infectious diseases in animals (1), such as fowl cholera in poultry (2), atrophic rhinitis in pigs (3), rabbit snuffles (4), and both pneumonia and hemorrhagic septicemia in ruminants (5). Among these diseases, acute hemorrhagic septicemia in cattle and sheep is particularly devastating and is characterized by rapid onset, high transmissibility, and a high mortality rate, thus causing significant economic losses in the livestock industry (6–8). Given the serious threat posed by *P. multocida*, it is crucial to establish a rapid, sensitive, and field-deployable detection method that can facilitate early diagnosis and effective control of the infection, particularly in resource-limited settings.

Currently, the diagnosis of *Pasteurella multocida* and related diseases relies not only on conventional pathogen identification techniques (9, 10) but also on serological assays (11), immunohistochemistry (12), and molecular diagnostic methods (13, 14). Among these methods, polymerase chain reaction (PCR) is regarded as the gold standard for *P. multocida* detection because of its exceptional specificity and reliability (15). However, PCR typically requires specialized laboratory infrastructure and sophisticated equipment, limiting its applicability in field or low-resource settings.

In recent years, the development of isothermal amplification technologies has enabled molecular diagnostics to move beyond dependence on thermal cyclers, leading to faster, more sensitive, and more versatile detection platforms (16, 17). Recombinase polymerase amplification (RPA), developed in 2006, is a rapid and cost-effective nucleic acid amplification method that operates at a constant temperature of 37–42 °C, amplifying DNA within 10 to 30 min (18). It eliminates the need for expensive thermocyclers and has been widely applied in microbial diagnostics. However, traditional RPA readouts often rely on gel electrophoresis, which is time-consuming and labor-intensive and poses biosafety concerns—factors that limit its practicality for point-of-care use (14). This integrated system has demonstrated high sensitivity and rapid, simple operation in detecting a variety of pathogens, including *Mycobacterium tuberculosis*, SARS-CoV-2, human papillomavirus (HPV) and *Staphylococcus aureus* (19–22). However, to our knowledge, a comprehensive RPA-CRISPR/Cas12a system specifically optimized for the visual detection of Pm has not yet been reported. Compared with conventional diagnostic methods, this integrated approach offers high sensitivity, a short turnaround time, and simple operation, presenting a highly promising solution for onsite detection of Pm in resource-limited settings.

To address these limitations, RPA has recently been integrated with other detection strategies, such as lateral flow dipsticks (LFDs) and CRISPR/Cas systems. The CRISPR/Cas system, which consists of clustered regularly interspaced short palindromic repeats (CRISPR) and CRISPR-associated (Cas) proteins, originated as an adaptive immune mechanism in bacteria and archaea, protecting them against invasive genetic elements (23). A key breakthrough was the discovery of the trans-cleavage activity of Cas12a. Upon activation, this enzyme nonspecifically cleaves single-stranded DNA molecules, a feature that has enabled the development of several RPA-CRISPR/Cas12a-based diagnostic assays (24), including platforms such as DETECTR (25). Compared with conventional diagnostic methods, this integrated system offers high sensitivity, a short turnaround time, and simple operation, resulting in a highly promising solution for onsite detection of *P. multocida* in resource-limited settings.

In this study, we developed a simple, sensitive, and visually interpretable rapid detection method by integrating recombinase polymerase amplification (RPA) with the CRISPR/Cas12a system. First, RPA primers targeting the kmt1 gene of *P. multocida* were designed and optimized for efficient /amplification. Candidate CRISPR RNAs (crRNAs) were subsequently screened, and the molar ratio between crRNA and Cas12a, as well as the reaction time for the combined RPA-Cas12a system, was systematically optimized. The sensitivity and specificity of the optimized assay were then evaluated. Finally, the feasibility of the method was assessed by testing 102 clinical samples suspected of *P. multocida* infection and comparing the results with those obtained from real-time PCR.

## 2 Materials and methods

### 2.1 Materials

The recombinase polymerase amplification (RPA) kit used for isothermal amplification was purchased from Amp-Future Biotech Co., Ltd. (Changzhou, China). RPA primers, crRNAs, and ssDNA probes were obtained from Tsingke Biotechnology Co., Ltd. (Beijing, China). EnGen® Lb Cas12a (Cpf1) was purchased from New England BioLabs (Beijing, China). The DNA extraction kit was obtained from ONOBATE Biotech Co., Ltd. (Xianyang, China).

### 2.2 Bacterial plasmids and clinical samples

A 457 bp conserved sequence of the Pm kmt1 gene obtained from the GenBank database was inserted into the pMD18-T vector and synthesized by Beijing Qingke Biotechnology Co., Ltd. Glycerol-preserved bacteria were revived, and the plasmids were extracted and sequenced to serve as positive controls for this study. A total of 102 nasal swab samples were collected from suspected Pm-infected livestock in various farming regions of Xinjiang, China. DNA was extracted from the samples, purified using standard protocols, and stored at −20 °C. PCR analysis revealed that 10 clinical samples were positive for Pm infection, whereas 92 samples were negative.

### 2.3 RPA primer design

RPA primers targeting the Pm kmt1 gene were designed according to RPA primer design principles. The sequences of the primer pairs are shown in Table 1. Primer Premier 5.0 software was used to analyze the hairpin structures and dimers of the primers. The specificity of the RPA primers was evaluated using Primer-BLAST from NCBI. Primer validation was performed using the synthetic DNA template of the Pm kmt1 plasmid.

**Table 1:** Primers and crRNA designed in this study.

**Primer/crRNA**	**Sequences 5′-3′**	**Length**
1 kmt1-F	GATTGGCTCAACACACCAAACTCCGCCCAACA	32 nt
1 kmt1-R	GATTGCCGCGAAATTGAGTTTTATGCCACTTGAA	33 nt
2 kmt1-F[22]	TATGGCTCGTTGTGAGTGGGCTTGTCGGTAGT	31 nt
2 kmt1-R[22]	TAAATAACGTCCAATCAGTTGCGCCGTTGTCAAG	33 nt
crRNA 1	UAAUUUCUACUAAGUGUAGAUCCACACGCCAAAUAAAGACU	41 nt
crRNA 2	UAAUUUCUACUAAGUGUAGAUGCGUGUGGCAAAGAAAAGCA	41 nt
crRNA 3	UAAUUUCUACUAAGUGUAGAUUUUGCCACACGCCAAAUAAA	41 nt

### 2.4 Reaction design and optimization of RPA

RPA reactions were performed using a recombinase polymerase amplification kit (Amp-Future Biotech Inc., Changzhou, China). Briefly, 14.7 μL of buffer A, 1 μL of kmt1-F, 1 μL of kmt1-R, and 5 μL of ddH₂O were added to the lyophilized reaction tube and mixed. Afterward, 2 μL of the kmt1 template and 1.3 μL of buffer B were added to initiate the reaction. Upon completion of the reaction, DNA extraction buffer was added at a 1:1 ratio, followed by centrifugation at 12,000 rpm for 5 min. A 5 μL aliquot of the supernatant was loaded onto a 2% agarose gel for electrophoretic validation of the RPA products.

Several parameters were compared to optimize the RPA reaction conditions. First, optimal primer pairs were selected by comparing the amplification efficiency of two different primer sets. Additionally, amplification efficiency may be influenced by the reaction time and incubation temperature. Thus, the reaction mixtures were incubated at six different temperatures (35 °C, 36 °C, 37 °C, 38 °C, 39 °C, and 40 °C) for six different durations (5 min, 10 min, 15 min, 20 min, 25 min, and 30 min). After the reactions were completed, the amplified products were analyzed via agarose gel electrophoresis, and the optimal conditions were determined based on the brightness of the DNA bands.

### 2.5 Design of CrRNA and ssDNA

In the CRISPR/Cas12a system, the activation of Cas12a relies on PAM recognition and target DNA binding. In accordance with design principles, crRNAs for Lb Cas12a were designed to target regions near the PAM site of the Pm kmt1 sequence. The crRNA sequences are listed in Table 1.

When the crRNA recognizes the target Pm kmt1 sequence and activates the Lb Cas12a protein, Cas12a indiscriminately cleaves DNA in the reaction system. On this basis, a short fluorescently labeled ssDNA fragment (probe) was added to the reaction system. During cleavage by Lb Cas12a, the fluorescent and quencher groups of the probe were separated, generating a fluorescence signal. The ssDNA probe sequence was 5’-FAM-TTATT-BHQ1-3′. After cleavage, green fluorescence could be observed under a blue-light transilluminator. The probe was synthesized by Beijing Qingke Biotechnology Co., Ltd., and stored at −20 °C.

### 2.6 Reaction design and optimization of RPA-CRISPR/Cas12a

The CRISPR/Cas12a reaction system consisted of 1 μL of Lb Cas12a protein, 2 μL of 10 × NEBuffer™ r 2.1, 1 μL of crRNA, 2 μL of ssDNA probe, 10 μL of nuclease-free water, and 4 μL of RPA product, resulting in a final volume of 20 μL. The reactions were performed at 37 °C according to the instructions provided with the Lb Cas12a protein.

To increase the specificity of crRNA-guided Cas12a activity and reduce off-target effects, three crRNAs were designed based on the RPA-amplifiable sequences. Cas12a reactions were conducted without interference from other variables, and the optimal crRNA was selected based on fluorescence intensity. The concentration ratio between Cas12a and crRNA is a key factor affecting the efficiency of the CRISPR-Cas12a reaction. Because different ratios produce varying effects, optimization of their concentrations was performed. Orthogonal experiments were conducted using different concentrations of the Cas12a protein (10 nM, 15 nM, 20 nM, 25 nM, and 50 nM) and crRNA (100 nM, 150 nM, 200 nM, 250 nM, 300 nM, 350 nM, and 500 nM) to determine the optimal ratio.

### 2.7 Specificity and sensitivity tests

DNA extracted from Mo, Mannheimia hemolytica, bovine coronavirus, *Staphylococcus aureus*, *Escherichia coli*, and Streptococcus was used as a template to evaluate the specificity of the reaction. To determine the sensitivity of the RPA-CRISPR/Cas12a reaction, the Pm standard plasmid was serially diluted tenfold for testing. Sensitivity was determined by observing fluorescence signals in the tube with the lowest DNA concentration. The DNA copy number was calculated using the following formula: Copies = [6.02 × 10^23^ × plasmid concentration (g/mL)]/plasmid molecular weight (g/mol). The RPA products were then subjected to CRISPR/Cas12a detection, with each reaction performed in triplicate.

### 2.8 Clinical sample detection

The PCR method was developed based on primer design according to T/SDAA (0045–2021) for sample testing. Ten PCR-positive and 92 PCR-negative samples were selected and tested using the established RPA-CRISPR/Cas12a method. The results of both methods were compared in parallel to evaluate their concordance.

## 3 Results

### 3.1 Optimization of the recombinase polymerase amplification reaction with pm recombinase

**Figure 1:**
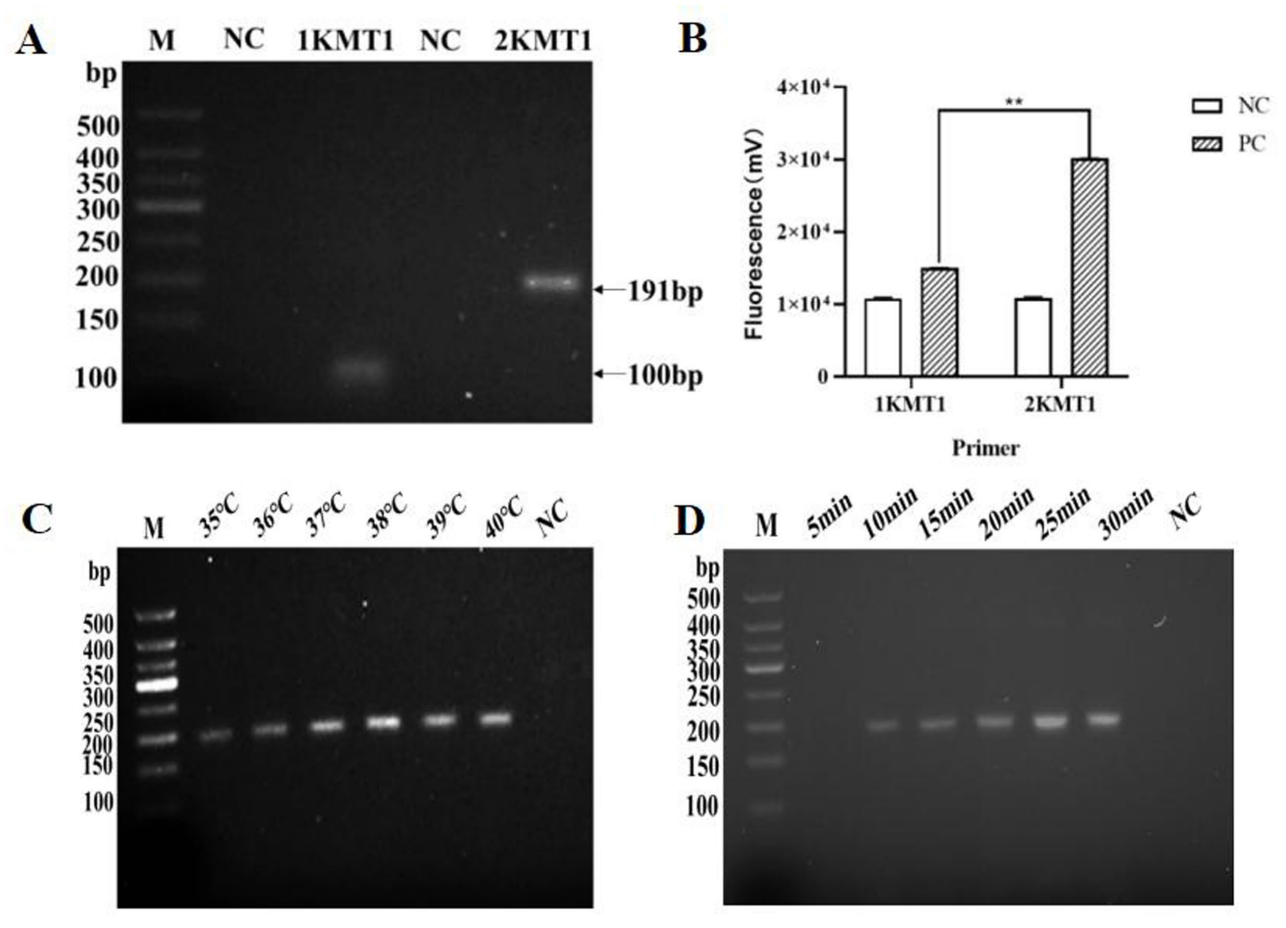
Optimization results of Pm recombinase polymerase amplification reaction **(A)** Gel electrophoresis results of RPA amplification with two pairs of primers; **(B)** Grayscale analysis of the resultant plots using ImageJ software; **(C)** RPA optimal reaction temperature screening; **(D)** RPA optimal response time screening (*p < 0.05; **p < 0.01; NC, negative control).

Using the standard plasmid as a template and nuclease-free water as the negative control, RPA amplification was performed with two different primer pairs. Primer selection was based on the brightness and clarity of the target bands in the gel electrophoresis results. As shown in Figure 1A, the amplification products matched the expected sizes: 101 bp for 1 kmt1 and 191 bp for 2 kmt1. Grayscale analysis revealed a significant difference between 2 kmt1 and 1 kmt1, leading to the selection of 2 kmt1 for subsequent experiments (Figure 1B). We also investigated the optimal reaction temperature for Pm RPA and reported that the band intensity increased from 35 °C to 38 °C, peaking at 38 °C, and then decreased at 39 °C and 40 °C (Figure 1C). Therefore, 38 °C was selected as the optimal temperature for RPA amplification in this study. Additionally, we examined the optimal reaction time and found that bands began to appear at 10 min and became more defined with increased time. Considering the need for rapid detection, the optimal reaction time was set at 15 min (Figure 1D).

### 3.2 Optimization of RPA-CRISPR/Cas12a

**Figure 2:**
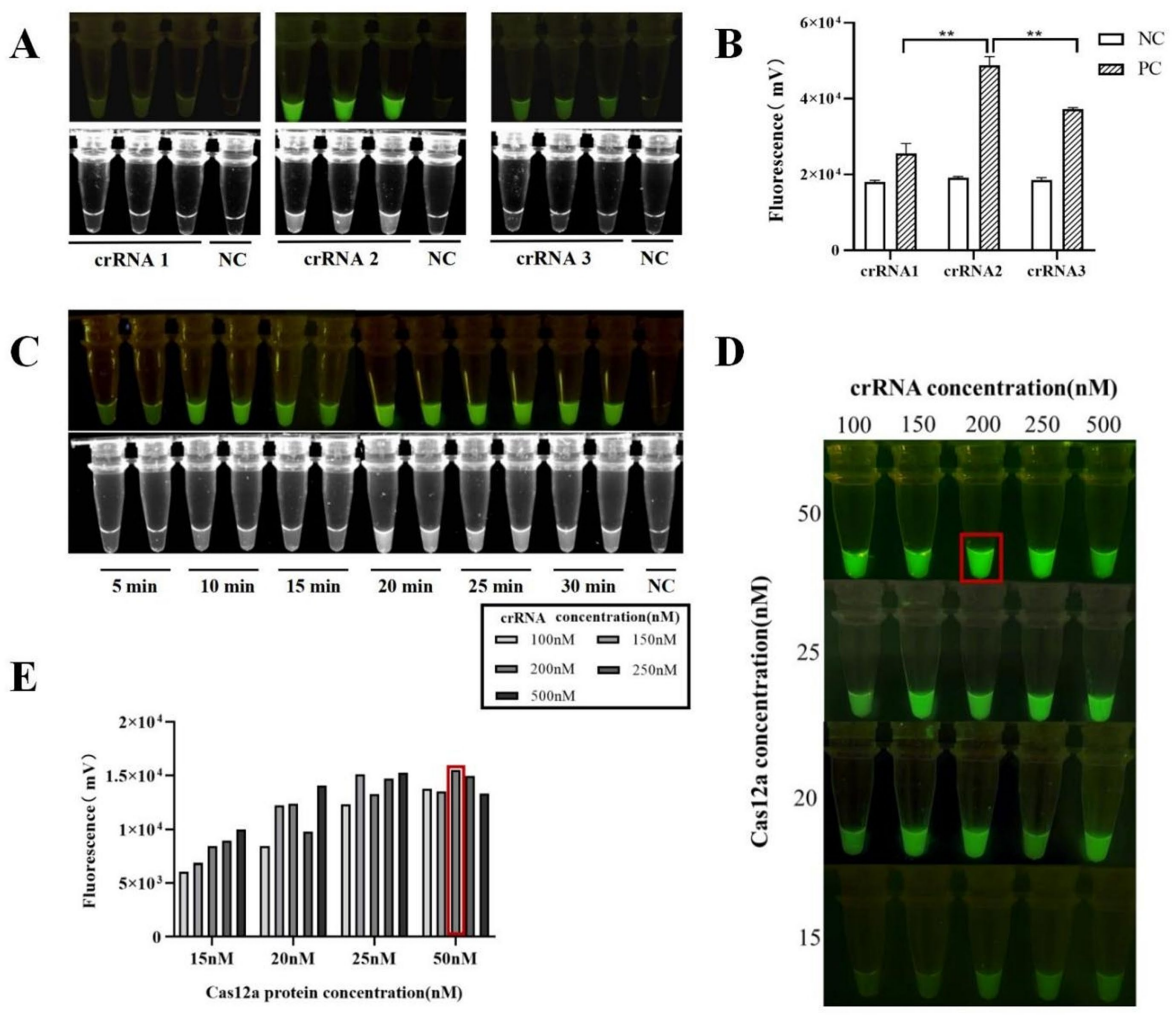
Optimization results of RPA-CRISPR/Cas12a **(A,B)** screening of crRNA sequences; **(C)** results of time optimization; **(D,E)** results of Cas12a/crRNA concentration optimization (*p < 0.05; **p < 0.01; NC, negative control).

Three designed crRNAs were tested to select the one that enabled the Cas12a protein to achieve maximum cleavage efficiency. Under a blue-light transilluminator and gel imaging system, compared with crRNA 1 and crRNA 3, crRNA 2 produced stronger fluorescence across the three replicates (Figure 2A), and grayscale analysis revealed statistically significant differences (Figure 2B). Therefore, crRNA 2 was selected as the optimal crRNA. To determine the optimal reaction time, a reaction system using 10^6^ copies/μL standard plasmid was incubated at 37 °C for durations ranging from 5 to 30 min. Weak fluorescence was visible after 5 min, and by 10 min, fluorescence was easily visible to the naked eye under the blue-light transilluminator and gel imaging system. To improve the detection accuracy, the reaction time was finalized at 15 min (Figure 2C). To optimize the concentration ratio, orthogonal experiments were conducted using gradients of Cas12a and crRNA concentrations, with fluorescence observed under a blue-light device to determine the optimal ratio. The fluorescence intensity generally increased with increasing concentrations of crRNA and Cas12a. When the Cas12a/crRNA concentrations were 50 nM/500 nM, the fluorescence was weaker than that at 50 nM/200 nM and 25 nM/500 nM, indicating the need for concentration optimization. Based on the principle of low-cost rapid detection, a 50 nM/200 nM ratio of Cas12a/crRNA was selected for subsequent reactions (Figures 2D,E).

### 3.3 Specificity analysis

**Figure 3:**
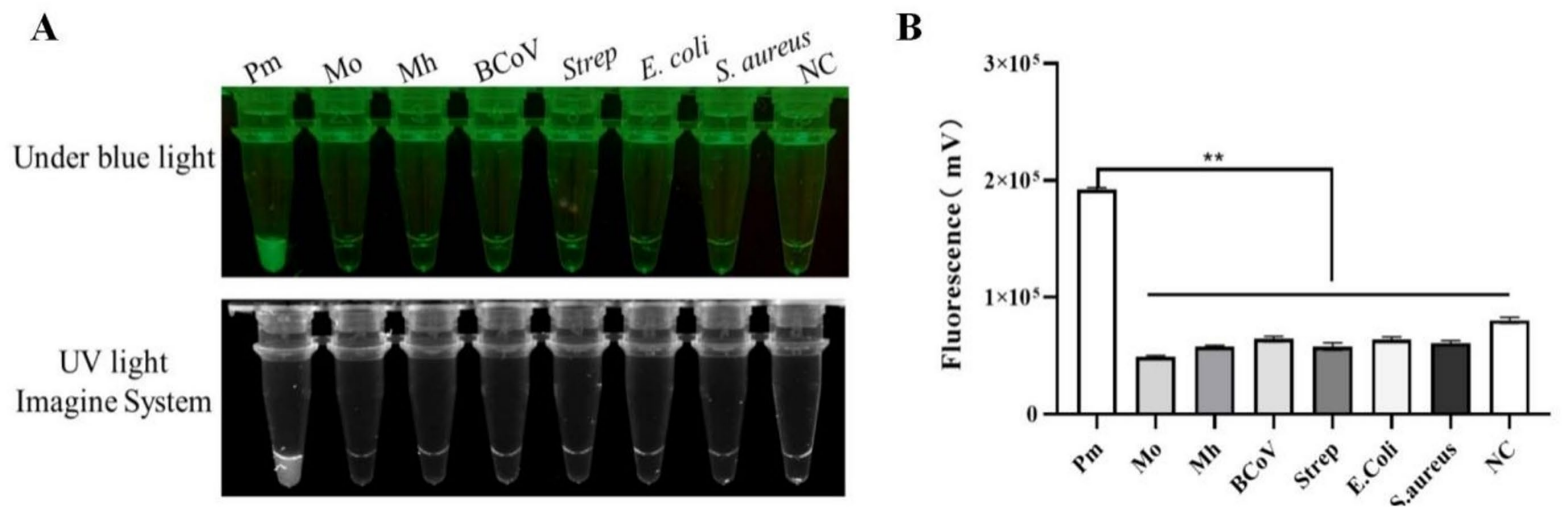
Results of specificity testing **(A)** Pm fluorescence intensity diagram; **(B)** Pm grayscale analysis diagram (*p < 0.05; **p < 0.01; NC, negative control).

To evaluate the specificity of the optimized RPA-CRISPR/Cas12a detection method, cDNA or DNA from common respiratory pathogens, including Mo, Mh, BCoV, *E. coli*, *S. aureus*, and Streptococcus, was used as a template, with nuclease-free water used as the negative control. The results revealed fluorescence signals only for Pm, while all non-Pm samples tested negative, indicating 100% specificity of the method (Figure 3).

### 3.4 Sensitivity analysis

**Figure 4:**
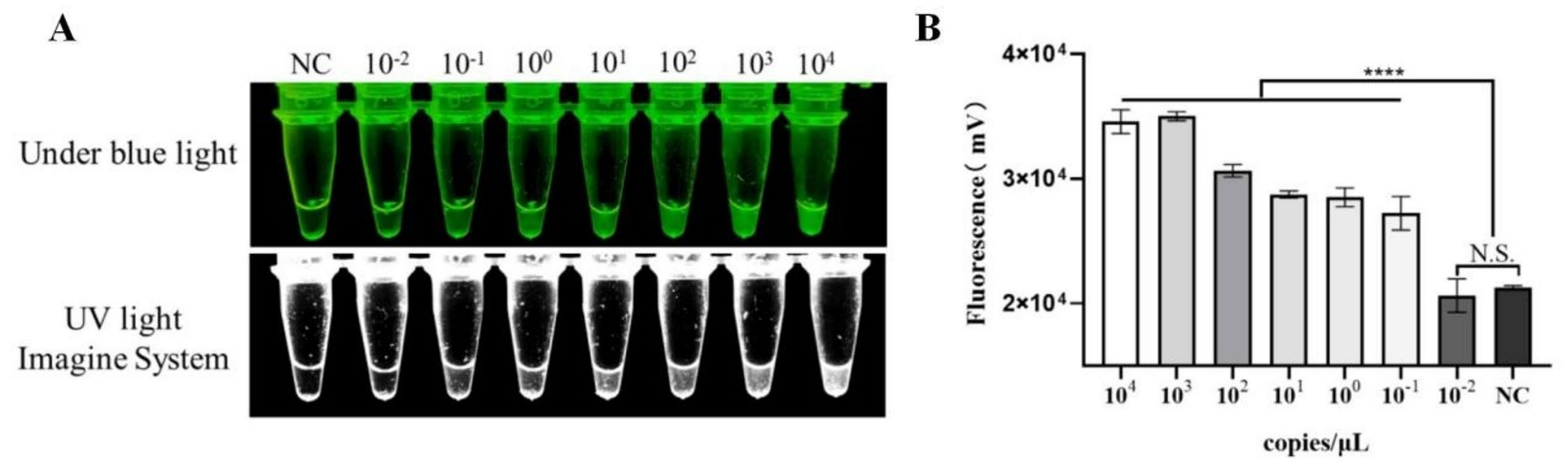
Results of sensitivity testing **(A)** PM fluorescence intensity diagram; **(B)** PM grayscale analysis diagram (*p < 0.05; **p < 0.01; NC, negative control).

The Pm-positive plasmid was serially diluted tenfold from 5 × 10^4^ to 5 × 10^−2^ copies/μL and tested using the RPA-CRISPR/Cas12a method, with nuclease-free water used as the negative control. At a plasmid concentration of 5 × 10^−2^ copies/μL, no visible fluorescence was observed, with no significant difference compared with the negative control. However, at a concentration of 5 × 10^−1^ copies/μL, weak fluorescence was detected, which was significantly different from that of the negative control, thus indicating that the detection limit of the established RPA-CRISPR/Cas12a method is 5 × 10^−1^ copies/μL (Figure 4).

### 3.5 Clinical sample detection

**Figure 5:**
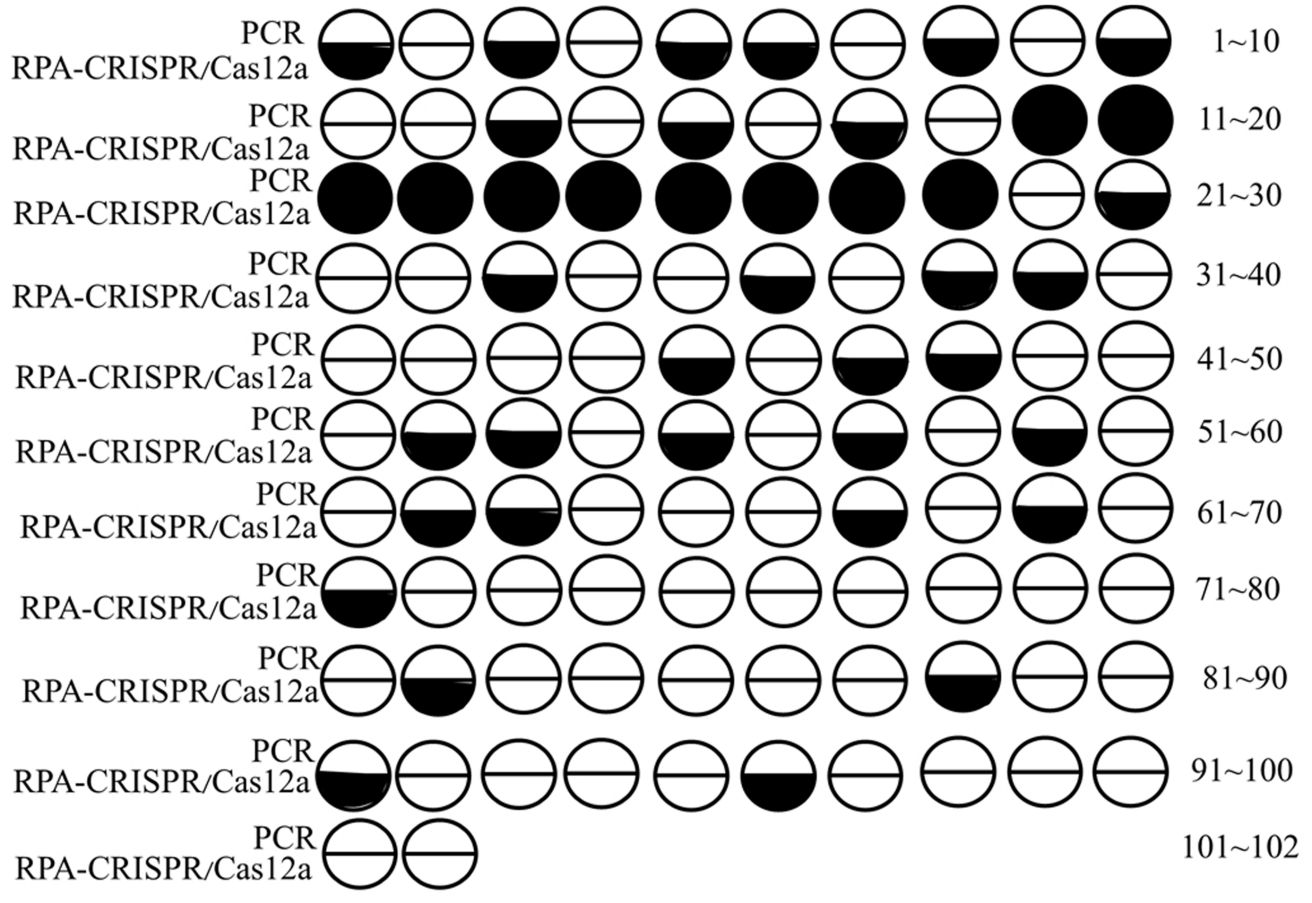
Results of the sample tests.

To evaluate the practical application of the RPA-CRISPR/Cas12a method for detecting Pm, 10 PCR-positive and 92 PCR-negative samples were tested using the RPA-CRISPR/Cas12a method. Portions of positive PCR and RPA products were also sequenced. The results revealed that the RPA-CRISPR/Cas12a method detected 41 positive samples, with a detection rate of 40.20% (Table 2). The concordance rate between the two methods was 100% (Figure 5), and all the sequencing results confirmed the presence of Pm.

**Table 2:** Test sample results.

Parameter	**PCR**	**RPA-CRISPR/Cas12a**
Positive/Total sample	10/102	41/102
Detection rate	9.80%	40.20%

## 4 Discussion

*Pasteurella multocida* (Pm), a pathogenic bacterium that can cause pneumonia, commonly infects sheep through exogenous routes, but endogenous infections may also occur when animal immunity is compromised. Infected sheep may exhibit mild symptoms such as dyspnea, diarrhea, progressive emaciation, and nasal discharge or may die from severe dehydration, septicemia, and pneumonia. In China, reports of this disease causing mortality in sheep—especially lambs—are frequently documented (26, 27). Currently, the gold standard for diagnosing Pm is PCR identification. Although this method is highly specific and reliable, it requires sterile laboratory conditions and expensive equipment, which limits its applicability for rapid field-based pathogen diagnosis. Therefore, developing a rapid, sensitive method suitable for field detection is highly important.

With the establishment of the DETECTR platform, combining RPA with CRISPR/Cas12a technology has opened new avenues for field diagnostics. By leveraging the nonspecific cleavage activity of Cas12a in the CRISPR/Cas12a system and introducing fluorescently labeled ssDNA into the reaction, visual detection can be achieved. The entire detection process is rapid and highly sensitive, and it eliminates the need for sterile laboratory environments or thermal cyclers, resulting in an ideal method for grassroots-level applications. In this study, a PCR method was designed on the basis of the T/SDAA (0045–2021) primer guidelines to investigate Pm infections in Xinjiang. The positive detection rate was 8.39% (13/155), confirming the presence and prevalence of the disease in sheep and underscoring the need for a rapid diagnostic method. Cao Yiheng conducted an epidemiological survey of large-scale sheep farms in five regions including Changji and Shihezi in Xinjiang, and reported a Pm nucleic acid positivity rate of 6.21% (18/290) (28). The detection rate in this study was slightly higher, possibly because only 30.34% (88/290) of the sheep sampled by Cao Yiheng had mild respiratory symptoms. Between 2019 and 2021, the Pm isolation rate among sheep with respiratory symptoms in Hejing County, Xinjiang, reached 40.8% (124/304) (29). Pathogen isolation yielded higher diagnostic rates than PCR did, which may be attributed—in addition to regional factors—to the lower sensitivity of PCR, especially in detecting low-copy nucleic acids without enrichment. Xu Tenglin et al. established a TaqMan real-time PCR method, which showed that its positive detection rate (40%) for clinical samples was significantly higher than that of the conventional PCR method (25%); this confirms the limitation of insufficient sensitivity of conventional PCR when detecting pathogens with low copy numbers (29).

RPA primers targeting the conserved region of the Pm kmt1 gene were designed, and an RPA reaction system was established through optimization of the reaction time and temperature. Experimental results revealed that detectable levels of nucleic acid amplification could be achieved within 10 min at 38 °C in a water or metal bath. To ensure more accurate detection, an amplification time of 15 min was selected in this study. PCR requires 90 min of thermal cycling between 94 °C and 55 °C, and factors such as reagent evaporation at high temperatures and variability in instrument quality may lead to false positives. Compared with PCR, the RPA method improves amplification efficiency by 83.33% and eliminates the need for large-scale instrumentation. RPA products are typically visualized via gel electrophoresis, but this requires protein extraction from the product, which is labor intensive and may result in excessive loss of low-concentration nucleic acids if not performed properly, leading to false-negatives. Additionally, the use of electrophoresis buffers and nucleic acid dyes adds safety risks that present challenges in diagnostic settings. With the discovery of the trans-cleavage activity of CRISPR/Cas12a, its combination with RPA has already been applied to detect other pathogens. In this study, the RPA product was used as the template to develop an RPA-CRISPR/Cas12a detection system. Optimization was conducted in terms of crRNA selection, reaction temperature, time, and Cas12a/crRNA concentration to determine the optimal conditions. The results indicate that the CRISPR/Cas12a method is capable of detecting RPA products within 15 min at 37 °C in a water or metal bath. This method obviates the need for extraction or electrophoresis to detect the RPA products, thus simplifying the workflow and preventing the loss of low-concentration nucleic acids during processing.

The RPA-CRISPR/Cas12a detection method enables visual detection at 37–38 °C within 30 min, with 100% specificity and a sensitivity of 10^−1^ copies/μL. Compared with that of the PCR method, its sensitivity is 10^7^ times greater, and the total detection time is reduced by 75%. In comparison to TaqMan real-time PCR technology, the sensitivity is increased 100-fold, and the detection time is shortened by over 50% (30). Both PCR and real-time PCR depend on thermal cycling systems and require specialized laboratory environments for detection. The LAMP method does not rely on large instruments and can complete detection at 65 °C within 60 min, with a detection limit of 10^2^ copies/μL (31). However, its reaction conditions and sensitivity still differ from those of the RPA-CRISPR/Cas12a method. In conclusion, the method is simpler and more sensitive than others, requires less time, and does not depend on large instruments, making it suitable for field-level detection.

Beyond the first application of the RPA-CRISPR/Cas12a system for Pm detection, this study provides substantial technical advancements to the existing methodology. Key optimizations include: (i) the rational design and screening of highly efficient crRNA specific to the Pm *kmt1* gene. (ii) the determination of an optimal Cas12a/crRNA molar ratio (50 nM/200 nM) that balances sensitivity and cost-effectiveness. (iii) the integration of RPA amplification at 38 °C with Cas12a detection at 37 °C within a streamlined 30-min protocol. These refinements collectively enhance the system’s sensitivity (down to 5 × 10^−1^ copies/μL), speed, and suitability for instrument-free field use, setting a valuable template for adapting RPA-CRISPR/Cas12a to other veterinary pathogens.

In recent years, the combined use of RPA and CRISPR/Cas systems has provided multiple solutions for the rapid on-site detection of Pm (14, 32). The RPA-CRISPR/Cas12a method established in this study, while inheriting the common advantages of this technical route such as rapidity, visibility, and no need for sophisticated instruments, has achieved significant performance improvements and application value innovations through the following aspects. Firstly, this study is the first to optimize this system for ovine Pm, filling the gap in on-site molecular diagnostic tools for this important host pathogen. Secondly, through systematic crRNA screening, Cas12a/crRNA molar ratio, etc., we have increased the detection sensitivity to 5 × 10^−1^ copies/μL, reaching an ultra-high level comparable to the most advanced research and significantly outperforming the traditional RPA-LFD method. Furthermore, the temperature control connection scheme we designed, which involves 38 °C RPA amplification and 37 °C Cas12a detection, enables the entire detection process to be completed within 30 min in a single constant-temperature device, with an extremely simple procedure. Compared with one-pot schemes that require special tube types or centrifugation steps, this scheme ensures ultra-high sensitivity while further reducing the requirements for operating equipment and consumables, achieving a better balance between cost, ease of use, and high performance, which is more in line with the actual conditions of grassroots farms. Finally, the detection rate of up to 40.2% in 102 clinical samples (4.1 times that of traditional PCR) strongly demonstrates the outstanding ability of this method in detecting latent or low-load infections, which is of great significance for precise prevention and control.

Based on primers from T/SDAA (0045–2021), a PCR method was designed for sample detection. Ten positive and 92 negative samples were tested clinically using the RPA-CRISPR/Cas12a method. The detection rate was 40.2%, which is 4.1 times higher than that of PCR. The two methods showed 100% concordance, and all the sequencing results confirmed the presence of Pm. These results further demonstrate the accuracy of the Pm RPA-CRISPR/Cas12a detection method and its suitability for clinical applications.

We will prepare simulated samples containing different concentrations of Pm target DNA using serum from healthy animals, eluates from nasal swab samples, to quantitatively assess the matrix inhibition effect and optimize the sample pretreatment (such as dilution, heating, or simple lysis) protocols to eliminate interference. In the laboratory, we will directly add inactivated Pm strains to real clinical sample matrices (such as serum, nasal swab sample homogenates) for comparative testing to evaluate the efficiency and robustness of the entire process from sample processing to final detection. We have initiated communication with our collaborating clinical units and plan to use archived or prospectively collected clinical samples with ethical approval in subsequent studies to compare this method with the existing gold standard methods to obtain real clinical sensitivity and specificity data (33–35).

## Data Availability

The original contributions presented in the study are included in the article/Supplementary material, further inquiries can be directed to the corresponding author/s.
