# Supplementary material for: RPA-CRISPR/Cas12a-based detection of Pasteurella multocida: establishment and initial application
Source: Front Vet Sci. 2026 Jan 14;12:1730229. doi: 10.3389/fvets.2025.1730229 (PMC12847050; doi:10.3389/fvets.2025.1730229)
Supplement: Supplementary file 1 [file Data_Sheet_1.PDF]

Supplementary Material

1 Supplementary Figures and Tables

1.1 Table 1. Primers and crRNA designed in this study.

Primer/crRNA	Sequences 5′-3′	Length
1 kmt1-F	GATTGGCTCAACACACCA AACTCCGCCCAACA	32 nt
1 kmt1-R	GATTGCCGCGAAATTGAG TTTTATGCCACTTGAA	33 nt
2 kmt1-F[22]	TATGGCTCGTTGTGAGTG GGCTTGTCGGTAGT	31 nt
2 kmt1-R[22]	TAAATAACGTCCAATCAG TTGCGCCGTTGTCAAG	33 nt
crRNA 1	UAAUUUCUACUAAGUGU AGAUCCACACGCCAAAU AAAGACU	41 nt
crRNA 2	UAAUUUCUACUAAGUGU AGAUGCGUGUGGCAAAG AAAAGCA	41 nt
crRNA 3	UAAUUUCUACUAAGUGU AGAUUUUGCCACACGCC AAAUAAA	41 nt

1.2 Table 2. Test sample results

	PCR	RPA-CRISPR/Cas12a
Positive/Total sample	10/102	41/102
Detection rate	9.80%	40.20%

1.3 Figure 1

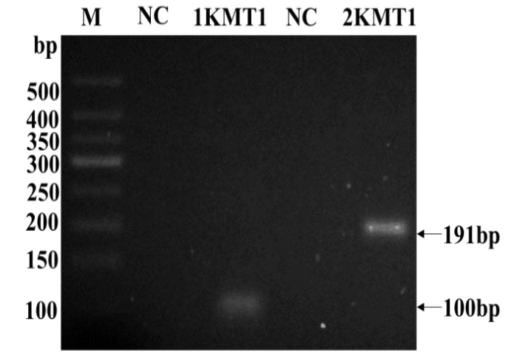

(A)

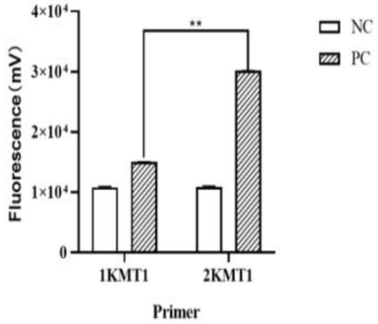

(B)

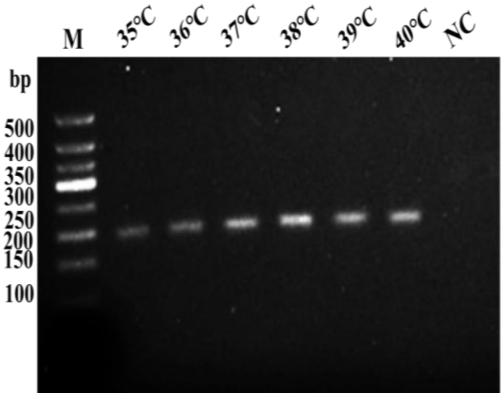

(C)

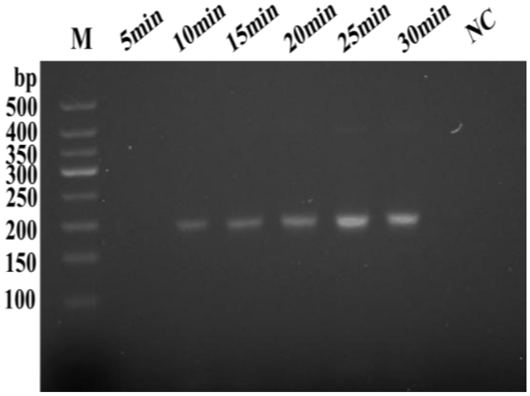

(D)

Figure 1. Optimization results of Pm recombinase polymerase amplification reaction (A) Gel electrophoresis results of RPA amplification with two pairs of primers.; (B) Grayscale analysis of the resultant plots using ImageJ software.; (C) RPA Optimal Reaction Temperature Screening.; (D) RPA Optimal Response Time Screening.（*：P < 0.05；**：P < 0.01；NC: Negative control）

1.4 Figure 2

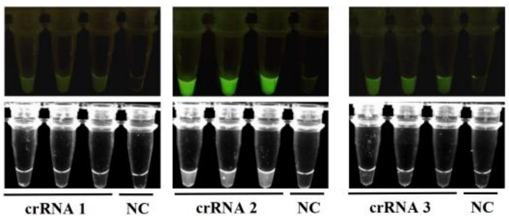

(**A**)

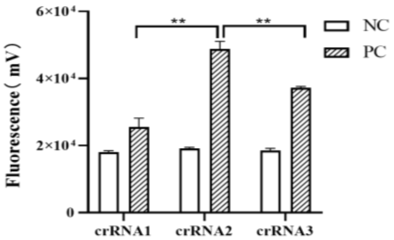

(**B**)

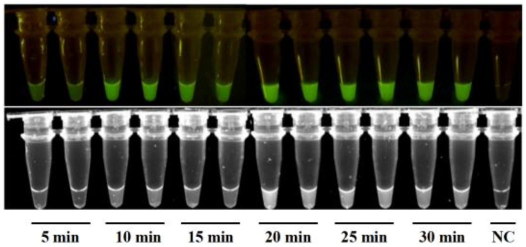

(C)

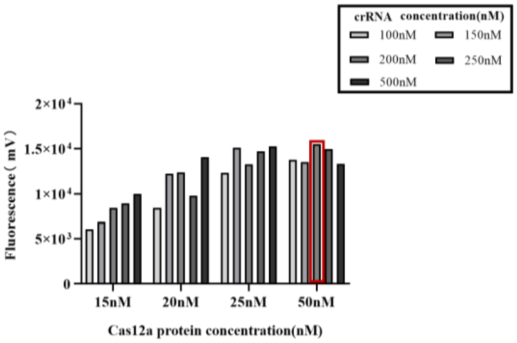

(E)

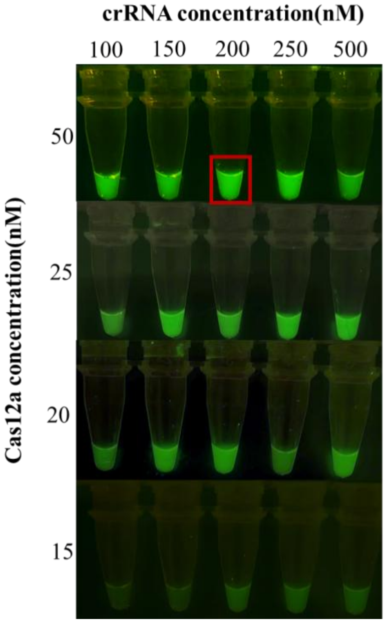

(D)

Figure 2. Optimization results of RPA-CRISPR/Cas12a (A、B) Screening of crRNA Sequences.; (C) Results of time optimization.; (D、E) Results of Cas12a/crRNA concentration optimization.（*：$P < 0.05$；**：$P < 0.01$；NC: Negative control）

1.5 Figure 3

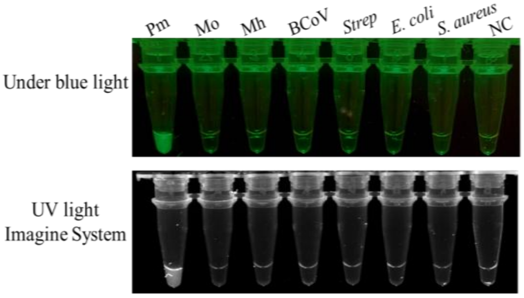

(A)

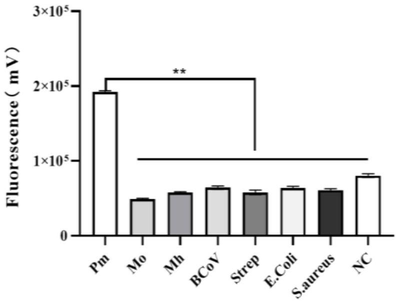

(B)

Figure 3. Results of specificity testing (A) Pm fluorescence intensity diagram.; (B) Pm grayscale analysis diagram.（*：$P < 0.05$；**：$P < 0.01$；NC: Negative control）

1.6 Figure 4

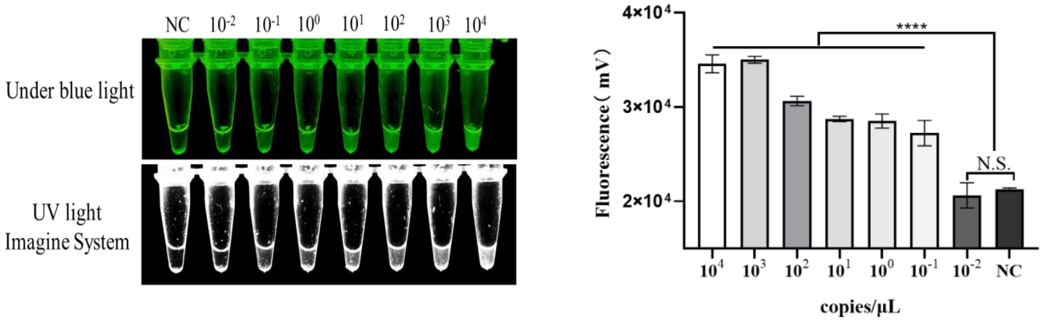

(**A**) (**B**)

Figure 4. Results of Sensitivity testing (A) Pm fluorescence intensity diagram.; (B) Pm grayscale analysis diagram.（*：P < 0.05；**：P < 0.01；NC: Negative control）

1.7 **Figure 5**

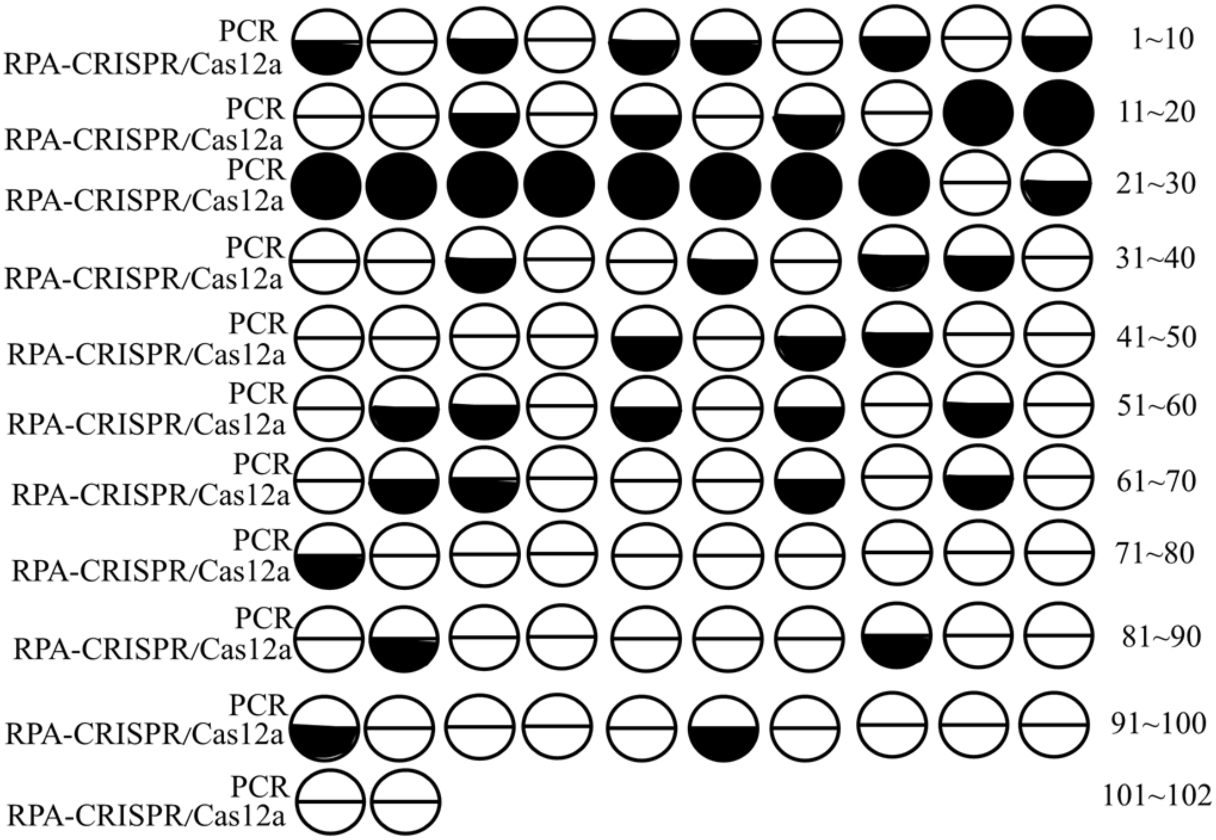

Figure 5. Results of the sample tests
